# Evaluation of the association between extravascular lung water and prognosis of sepsis: A protocol of systematic review

**DOI:** 10.1097/MD.0000000000023971

**Published:** 2021-02-05

**Authors:** Peng Luo, Cai-xia Song, Ye-qing Ai, Zhe Chen, Sheng-nan Yan, Xia Liu, Ying Chen, Jia-bin Sun

**Affiliations:** a Department of Cardio-thoracic Surgery, The First Affiliated Hospital of Jiamusi University, Jiamusi; b Department of Intensive Care Unit, The Central Hospital of Jinzhou City, Jinzhou; c Department of General Surgical Intensive Care Unit, The 910th Hosptial, Quanzhou; d Department of Intensive Care Unit, The First Affiliated Hospital of Jiamusi University, Jiamusi; e Department of Urological Surgery, Henan Second People's Hospital, Zhengzhou; f Department of Dermatology, The People's Hospital of Ningxia Hui Autonomous Region, Yinchuan, China.

**Keywords:** association, extravascular lung water, sepsis

## Abstract

**Background:**

The purpose of this study is to explore the association between extravascular lung water (EVLW) and prognosis of sepsis (PS).

**Methods:**

We will carry out comprehensive literature search in electronic databases (PUBMED/MEDLINE, EMBASE, CENTRAL, WorldSciNet, PsycINFO, Allied and Complementary Medicine Database, CBM, and CNKI) and additional sources. All electronic databases will be searched from their initial to the present without language restrictions. Case-controlled studies reporting the association between EVLW and PS will be evaluated for inclusion. Outcomes of interest will include mortality rate, extravascular lung water index, pulmonary vascular permeability index, blood lactate clearance, oxygenation index, blood gas analysis, PaO2/FiO2, cardiac output index, global end diastolic volume index, intrathoracic blood volume index, systemic resistance index, acute physiology and chronic health scoring system II, and infection-related organ failure scoring system. Study quality will be evaluated using Newcastle-Ottawa Tool, and statistical analysis will be performed utilizing RevMan 5.4 software.

**Results:**

This study will summarize the most recent evidence to investigate the association between EVLW and PS.

**Conclusions:**

The results of this study will provide an exhaustive view of the association between EVLW and PS.

**Study registration OSF:**

osf.io/vhnxw.

## 1 Introduction

Sepsis is a life-threatening organ dysfunction disorder that occurs when the dysregulated host response to an infection.^[1–4]^ It is a global health concern with high mortality and morbidity.^[1,5–7]^ The mortality rate of severe sepsis is about 25%, and that of septic shock is about 50%.^[8–10]^ It has been estimated that its incidence ranges from 20% to 50%.^[10]^ Other studies reported that its incidence has increased by 1.5% each year, and the number of attacked sepsis patients may be reach to 1 million in 2020.^[11–13]^ Studies found that extravascular lung water (EVLW) is associated with prognosis of sepsis (PS), resulting in subsequent organ dysfunction and increased mortality.^[14–26]^ However, there is restricted evidence to support the association between EVLW and PS. Thus, this study will systematically and comprehensively investigate the association between EVLW and PS.

## 2 Methods

### 2.1 Study registration

We have registered this study on OSF (osf.io/vhnxw). It is organized based on the guidelines of Preferred Reporting Items for Systematic Reviews and Meta-Analysis Protocol Statement.^[27]^

### 2.2 Eligibility criteria

#### 2.2.1 Types of studies

All case-controlled studies (CCSs) that reported the association between EVLW and PS will be considered for inclusion. Any other studies, except CCSs, will be excluded in this study.

#### 2.2.2 Types of exposures

Patients who were diagnosed as sepsis with EVLW were included in the experimental group.

Participants who were diagnosed as sepsis without EVLW were included as comparators.

#### 2.2.3 Types of participants

Patients with a clinical diagnosis of sepsis with or without EVLW will be included in this study regardless race, gender, and country.

#### 2.2.4 Types of outcome measurements

The outcomes consist of mortality rate, EVLW index, pulmonary vascular permeability index, blood lactate clearance, oxygenation index, blood gas analysis, PaO2/FiO2, cardiac output index (CI), global end diastolic volume index, intrathoracic blood volume index, systemic resistance index, acute physiology and chronic health scoring system II, and infection-related organ failure scoring system score.

### 2.3 Search strategy

We will comprehensively perform a broad literature search in both electronic databases (PUBMED/MEDLINE, EMBASE, CENTRAL, WorldSciNet, PsycINFO, Allied and Complementary Medicine Database, CBM, and CNKI) and additional sources (such as conference abstracts, clinical trial registries, and reference lists of included trials). We will search all sources from inception to the present without limitations to language. We will build an example of search strategy for CENTRAL in a Table 1. We will also modify similar search strategy for other electronic databases.

**Table 1:** Search strategy of CENTRAL.

Number	Search terms
1	MeSH descriptor: (sepsis) explode all trees
2	MeSH descriptor: (shock, septic) explode all trees
3	((pyemia^∗^) or (pyemias^∗^) or (pyohemia^∗^) or (septicemia^∗^) or (septicemias^∗^) or (blood poisoning^∗^) or (sepses^∗^) or (septic^∗^) or (endotoxemia^∗^)):ti, ab, kw
4	Or 1–3
5	MeSH descriptor: (extravascular lung water) explode all trees
6	((acute lung injury^∗^) or (acute respiratory distress syndrome^∗^) or (extravascular lung water^∗^) or (fluid management^∗^) or (fluid responsiveness^∗^) or (hemodynamic monitoring^∗^)or (lung oedema^∗^) or (pulmonary vascular permeability index^∗^) or (transpulmonary thermodilution^∗^)):ti, ab, kw
7	Or 5–6
8	MeSH descriptor: (case-control studies) explode all trees
9	MeSH descriptor: (observational studies as topic) explode all trees
10	((case-control^∗^) or (case referent^∗^) or (observational^∗^) or (trial^∗^) or (study^∗^) or (control^∗^)):ti, ab, kw
11	Or 8–10
12	4 and 7 and 11

### 2.4 Citation management and screening

Results from all searches in all sources will be imported to Endnote X9, and all duplicates will be excluded. Titles/abstracts will be scanned, and all irrelevant studies will be eliminated as appropriate. Then, full text articles will be checked cautiously against all eligibility criteria. The whole process of all citation selection will be performed by 2 independent authors. Any conflicts in the screening process will be solved by discussion with a third author involved. Reasons for all excluded studies will be recorded in a table. The process of study selection will be presented in a flow diagram.

### 2.5 Data extraction and management

Data will be extracted by 2 independent authors using predefined and standardized data extraction sheet. The following information will be extracted as follows:

(1) publication information and study methodology, such as title, first author, year of publication, study period, and sample size.
(2) participants characteristics, eligibility criteria, duration and severity of disease.
(3) outcomes, results, findings, and conflict of interest. Any divisions within data extraction will be settled down through discussion with the help of another author.

### 2.6 Study quality assessment

Study quality of CCSs will be appraised using Newcastle-Ottawa Tool.^[28]^ It cover 3 broad aspects of selection of study groups, comparability and ascertainment of outcome of interest. Two independent authors will appraise study quality of all eligible CCSs. Any confusions will be cleared up by a third author through discussion or consultation.

### 2.7 Measurements of treatment effect

For continuous outcome data, they will be calculated as standardized mean difference and 95% CIs. If necessary, we will also collect medians and ranges as needed. For dichotomous outcome data, they will be presented as odds ratio or risk ratio and 95% CIs.

### 2.8 Statistical heterogeneity assessment

We will assess statistical heterogeneity using *I*^*2*^ test. The degree of heterogeneity is considered as follows: *I*^*2*^ ≤25% means low heterogeneity; 25%<*I*^*2*^ ≤75% exerts moderate heterogeneity; and *I*^*2*^ >75% suggests remarkable heterogeneity.

### 2.9 Data synthesis

This study will employ RevMan 5.4 software for data analysis. A detailed description of patient characteristics, study methods, outcome indicators, and methodological quality will be reported in evidence tables and discussed in the text. Whenever necessary, data from individual trials will be pooled using a random-effects model, and we will carry out a meta-analysis based on the low heterogeneity across those studies, and limited variations in study information, patient information, and outcome indicators. If the heterogeneity is moderate, we will perform subgroup analysis to investigate its potential reasons. If the heterogeneity is obvious, and it can not be elaborated by clinical or methodological levels, outcome data will not be pooled, and a meta-analysis will not be undertaken. Instead, we will report study findings through a narrative synthesis.

### 2.10 Subgroup analysis

If sufficient data is extracted, we will carry out subgroup analysis based on the variations in study information, patient characteristics, and outcome indicators.

### 2.11 Sensitivity analysis

Sensitivity analysis will be performed to verify the stability of pooled outcome results by deleting low quality trials, and studies with sample size less than 10.

### 2.12 Publication bias

If over 10 eligible studies are entered in this study, we will check publication bias using funnel plot and Egger regression test.^[29,30]^

### 2.13 Ethics and dissemination

This study is a secondary analysis of published data, thus no ethical approval is needed. We plan to disseminate this study on a peer-reviewed journal or a conference meeting.

## 3 Discussion

Previous studies have reported the association between EVLW and PS. However, there is insufficient evidence-based medicine evidence to support it. This study will comprehensively collect all potential studies from both electronic databases and other literature sources. Eligible studies will be included after all record selection, and essential data will be extracted. Then, the association between EVLW and PS will be explored through investigating outcomes of mortality rate, EVLW index, pulmonary vascular permeability index, blood lactate clearance, oxygenation index, blood gas analysis, PaO2/FiO2, CI, global end diastolic volume index, intrathoracic blood volume index, systemic resistance index, acute physiology and chronic health scoring system II, and infection-related organ failure scoring system score. The results of this study will provide helpful evidence base to the clinical practice and future researches.

## Author contributions

**Conceptualization:** Peng Luo, Ye-qing Ai, Zhe Chen, Sheng-nan Yan, Xia Liu, Ying Chen, Jia-bin Sun.

**Data curation:** Peng Luo, Cai-xia Song, Ye-qing Ai, Zhe Chen, Sheng-nan Yan, Ying Chen, Jia-bin Sun.

**Formal analysis:** Peng Luo, Cai-xia Song, Sheng-nan Yan, Xia Liu, Jia-bin Sun.

**Funding acquisition:** Jia-bin Sun.

**Investigation:** Jia-bin Sun.

**Methodology:** Peng Luo, Cai-xia Song, Ye-qing Ai, Zhe Chen, Sheng-nan Yan, Xia Liu.

**Project administration:** Jia-bin Sun.

**Resources:** Peng Luo, Cai-xia Song, Ye-qing Ai, Zhe Chen, Sheng-nan Yan, Xia Liu, Ying Chen.

**Software:** Peng Luo, Cai-xia Song, Ye-qing Ai, Zhe Chen, Sheng-nan Yan, Xia Liu, Ying Chen.

**Supervision:** Jia-bin Sun.

**Validation:** Peng Luo, Cai-xia Song, Sheng-nan Yan, Xia Liu, Ying Chen, Jia-bin Sun.

**Visualization:** Peng Luo, Cai-xia Song, Ye-qing Ai, Zhe Chen, Sheng-nan Yan, Xia Liu, Ying Chen, Jia-bin Sun.

**Writing – original draft:** Peng Luo, Ye-qing Ai, Zhe Chen, Xia Liu, Ying Chen, Jia-bin Sun.

**Writing – review & editing:** Peng Luo, Cai-xia Song, Zhe Chen, Sheng-nan Yan, Xia Liu, Ying Chen, Jia-bin Sun.
